# Histopathological growth patterns of neuroendocrine tumor liver metastases

**DOI:** 10.1007/s10585-023-10211-z

**Published:** 2023-05-14

**Authors:** Y. Meyer, A. Bohlok, P. Olthof, V. Donckier, M. Doukas, V. Lucidi, P. Vermeulen, D. Grünhagen, C. Verhoef

**Affiliations:** 1 Department of Surgical Oncology and Gastrointestinal Surgery, Erasmus University Hospital, Erasmus MC Cancer Institute, Rotterdam, The Netherlands; 2 Institut Jules Bordet, Surgical Oncology, Université Libre de Bruxelles, Brussels, Belgium; 3 Department of Pathology, Erasmus MC Cancer Institute, Rotterdam, The Netherlands; 4 Hôpital Erasme, Department of Abdominal Surgery, Université Libre de Bruxelles, Brussels, Belgium; 5 Translational Cancer Research Unit (GZA Hospitals and University of Antwerp), Antwerp, Belgium

**Keywords:** Neuroendocrine tumour liver metastases, Histopathological growth patterns, Prognosis, Prediction

## Abstract

Histopathological growth patterns (HGPs) of liver metastases represent a potential biomarker for prognosis after resection. They have never been studied in neuroendocrine tumor liver metastases (NETLM). This study evaluated if distinct HGPs can be observed in resected NETLM and if they have prognostic value. Sixty-three patients who underwent resection of NETLM between 01–01-2001 and 31–12-2021 were retrospectively included. HGPs were scored on Haematoxylin&Eosin slides using light microscopy, distinguishing desmoplastic- (dHGP), pushing- (pHGP) and replacement HGP (rHGP). Average HGP scores were calculated per patient. Each patient was classified according to predominant HGP. Overall and Disease-Free Survival (OS and DFS) were evaluated through Kaplan–Meier analysis and Cox regression. Eighteen patients had predominant dHGP (29%), 33 had predominant pHGP (52%) and 11 had predominant rHGP (17%). One patient had mixed HGP (2%). Five-year OS was 76% (95%CI: 66–87%) for the overall cohort. Five-year OS was 92% (95%CI: 77–100%) for dHGP, was 73% (95%CI: 59–91%) for pHGP, 50% (95%CI: 25–100%) for rHGP. Five-year DFS was 39% (95%CI: 19–83%) for dHGP, 44% (95%CI: 27–71%) for rHGP and 50% (95%CI: 23–100%) for pHGP. There was no significant association between HGP and OS or DFS in multivariable analysis. Distinct HGPs could be identified in NETLM. In patients who underwent resection of NETLM, no association was found between HGPs and postoperative survival. Half of the patients with NETLM have a predominant pushing growth pattern, which is a rare growth pattern in liver metastases from breast and colorectal cancer.

## Introduction

Histopathological Growth Patterns (HGPs) represent a promising prognostic biomarker in patients who underwent resection of liver metastases [1]. HGPs are evaluated at the tumor-liver interface (TLI) and classified into distinct categories. Desmoplastic HGP (dHGP) is characterized by a fibrous rim that separates the tumor cells from the surrounding liver parenchyma. There is no direct contact between the tumor cells and the hepatocytes. In replacement HGP (rHGP), the tumor cells seem to replace the hepatocytes in the surrounding liver cell plates, with direct contact between tumor cells and hepatocytes. The architecture of the liver is preserved. Pushing HGP is characterized by a sharp demarcation between the tumor and the surrounding liver parenchyma, without desmoplastic rim separating cancer cells and hepatocytes and without invasion of cancer cells into the surrounding liver parenchyma. The tumor does not preserve the liver architecture in pHGP [1, 2]. The association between HGPs and prognosis has been described most extensively in colorectal cancer liver metastases (CRLM). Patients with CRLM with a pure dHGP have significantly better overall survival (OS) and disease-free survival (DFS) after curative intent resection as compared to those with any rHGP component at the TLI [1, 3, 4]. HGPs may also predict the response to adjuvant chemotherapy in CLRM [5].

The prognostic value of HGPs has also been identified for liver metastasis from melanoma and breast cancer, as well as several other more rare kinds of liver metastases [6–8]. This suggests that, to some extent, the biological significance of HGPs could be independent of the primary tumor origin. Currently, much of the driving mechanisms behind HGPs remain unknown [1].

The HGPs of neuroendocrine tumor liver metastases (NETLM) have not been described yet. The liver is the most common organ involved in metastatic neuroendocrine tumors (NET). Due to the indolent course of the disease, NETLM are associated with a favorable prognosis compared to liver metastases from other malignant tumors [9]. Curative intent local treatment is recommended for treatment of NETLM with no extrahepatic metastatic disease and for symptom control in patients with functioning NETs. While OS is high after resection of NETLM, up to 85% in selected patients, there is a large variation in reported survival outcomes and postoperative recurrences are frequent [10, 11]. New biomarkers could improve patient selection and treatment outcomes. There are various treatment modalities available for NETLM. Local treatment for NETLM usually consists of surgical resection sometimes in combination with other local modalities like ablation. The use of local treatment is dependent on technical factors (for example: complete resection feasible and sufficient future liver remnant), patient factors, and prognostic factors. Important prognostic characteristics are tumour grade, morphology and KI-67 index. Upfront local treatment is generally reserved for grade 1–2 tumours, provided that the other criteria for resectability have been met. Most studies recommend systemic treatment in patients with grade 3 tumours [10, 12]. Liver transplantation is an option for local treatment for selected patients with unresectable NETLM, with favourable chacateristics [9, 13, 14]. For patients with somatostatin receptor positive NETLM there is also the option of Peptide Receptor Radionuclide Therapy [9, 15]. New biomarkers may help to select the most effective treatment for individual patients. In addition, the evaluation of HGPs in NETLM may be interesting from a biological perspective to provide a more complete overview of HGPs in secondary liver tumors. The goal of this study was to characterize the HGPs of NETLM and to evaluate their association with postoperative survivals in patients who underwent partial hepatectomy.

## Methods

A retrospective multicenter cohort study was conducted to describe the HGPs of NETLM. Patients were identified from the pathology records of the Erasmus University Medical Center (Rotterdam the Netherlands) and the Institut Jules Bordet, Université Libre de Bruxelles (Brussels, Belgium).

All patients who underwent curative intent resection for NETLM, which was defined as local treatment of all preoperatively identified metastatic lesions, were included. Patients with an unknown primary tumor who had undergone local treatment of all metastatic lesions, but not the primary tumor, were regarded as treated with curative intent.

All patients underwent local treatment between January 1st 2001 and December 31st 2021.

Clinicopathological data were retrospectively collected from the electronic patient records.

This study was approved by the institutional review board from both institutions (MEC 2020–0294, P2019/232)).

HGPs were assessed on Hematoxylin & Eosin (H&E) slides via light microscopy. All available slides were assessed for each patient. Each HGP was classified as a proportion of the TLI of each H&E slide. Slides were not assessed if their quality was insufficient for analysis or if there was no vital tumor.

The average HGP proportions were calculated per lesion and per patient to arrive at a single patient-level HGP. The patient level HGP was classified according to the updated 2022 consensus guidelines for scoring HGPs, which use 100% dHGP versus any amount of non-dHGP [1]. In addition, the HGPs were classified according to the predominant HGP, defined as the HGP present at > 50% of the TLI per patient, for explorative reasons. This second classification originates from the previous consensus guidelines for scoring HGPs [16].

Categorical data were reported as numbers and percentages. Continuous data were reported as median with interquartile range, unless specified otherwise. Categorical data were assessed using the chi-squared test, medians were compared using the Mann–Whitney U test for comparisons between two groups or the Kruskall-Wallis test for comparisons between multiple groups.

Overall and Disease Free Survival estimates were calculated using the Kaplan–Meier method and compared via log-rank test. The median follow-up for survivors was estimated using the reverse Kaplan–Meier method.

Cox regression for OS and DFS were corrected for WHO grade and origin of the primary tumor. Both covariates were chosen because they are known to be important prognostic factors in NETLM. No forward or backward selection for additional covariates was applied due to a lack of events for both OS and DFS.

OS was defined as the time in months from liver resection for NETLM to last follow-up or death. DFS was defined as the time in months from liver resection for NETLM to recurrence of disease, regardless of location or death.

Missing data was addressed via pairwise deletion.

All statistical analyses were conducted in R version 4.0.2 [17]. A two-sided p value of < 0.05 was considered statistically significant.

## Results

**Fig. 1:**
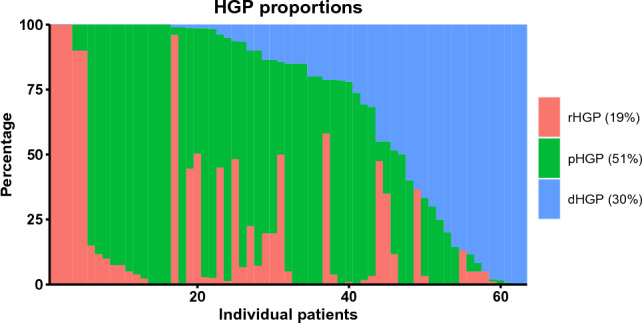
Distribution of HGP proportions per patient for the study cohort

HGPs were scored for all 63 patients. The patient-level proportions of observed HGPs are shown in Fig. 1. Classification using 100% dHGP versus any non-dHGP was not possible due to the rarity of pure dHGP in the current sample. Using the predominant classification eighteen patients had a predominant dHGP (29%), 33 had a predominant pHGP (52%) and 11 had a predominant rHGP (17%). There was one patient with a mixed HGP (2%).

Table 1 shows the baseline clinicopathological characteristics for each predominant HGP. No statistically significant differences were observed between the groups. Most patients had liver metastases with a low to intermediate tumour grade. That were well differentiated. There was a trend towards higher tumour grade in patients with rHGP (30%) compared to 20% in pHGP and 12% in dHGP. None of these differences were statistically significant. There was no difference in the amount of recurrences after liver resection between the groups (p = 0.751), with recurrence rates of 61% for dHGP, 71% for pHGP, and 60% for rHGP. The liver was the most common site of recurrence in all groups.

**Table 1:** Baseline characteristics per HGP (predominant)

		dHGP	pHGP	rHGP	Mixed HGP	p	Missing
n		18	33	11	1		
Sex (%)	Female	10 (56)	8 (25)	4 (36)	1 (100)	0.096	2.6
CCI (median [IQR])		7.5 [6.0, 9.5]	7.0 [7.0, 8.0]	7.0 [6.5, 8.0]	7.0 [7.0, 7.0]	0.966	3.9
Neoadjuvant PT (%)		1 (6)	8 (26)	3 (30)	0 (0)	0.309	9.1
Adjuvant PT (%)		3 (19)	5 (18)	2 (20)	0 (0)	0.969	13
Neoadjuvant LM(%)		2 (11)	9 (29)	3 (30)	0 (0)	0.262	5.2
Adjuvant LM (%)		5 (29)	5 (29)	5 (20)	2 (20)	0.805	15.6
Treatment PT (%)	None	8 (44)	8 (29)	3 (30)	0 (0)	0.62	10.4
	Resection	9 (50)	20 (71)	7 (70)	1 (100)		
	PRRT	1 (6)	0 (0)	0 (0)	0 (0)		
Origin PT (%)	Unknown	7 (39)	7 (23)	4 (36)	0 (0)	0.747	3.9
	Pancreas	5 (28)	10 (32)	1 (9)	0 (0)		
	Gastric	1 (6)	0 (0)	1 (9)	0 (0)		
	Small intestine	4 (22)	7 (23)	4 (36)	1 (100)		
	Thyroid	0 (0)	1 (3)	0 (0)	0 (0)		
	Colon	0 (0)	4 (13)	1 (9)	0 (0)		
	Lung	0 (0)	2 (6)	0 (0)	0 (0)		
	Mesentery	1 (6)	0 (0)	0 (0)	0 (0)		
Grade PT (%)	Low (G1)	1 (17)	9 (50)	2 (50)	0 (0)	0.62	54.5
	Intermediate (G2)	3 (50)	7 (39)	2 (50)	1 (100)		
	High (G3), small cell	2 (33)	1 (6)	0 (0)	0 (0)		
	High (G3), large cell	0 (0)	1 (6)	0 (0)	0 (0)		
Mitoses PT (%)	< 2 per 10 HPF	1 (25)	4 (50)	0 (0)	0 (NaN)	0.624	79.2
	2–20 per 10 HPF	2 (50)	2 (25)	1 (100)	0 (NaN)		
	> 20 per 10 HPF	1 (25)	2 (25)	0 (0)	0 (NaN)		
Ki67 PT (%)	< 3%	5 (56)	11 (65)	2 (67)	0 (0)	0.848	51.9
	3–20%	3 (33)	5 (29)	1 (33)	1 (100)		
	> 20%	1 (11)	1 (6)	0 (0)	0 (0)		
Differentiation PT(%)	Well Differentiated	5 (71)	13 (93)	2 (100)	0 (NaN)	0.095	61
pN stage (%)	N +	5 (71)	15 (79)	2 (50)	0 (0)	0.274	46.8
Functioning Tumor (%)		3 (19)	8 (27)	1 (11)	0 (0)	0.706	11.7
Metastases (%)	Synchronous	9 (50)	16 (53)	4 (36)	0 (0)	0.561	6.5
	Metachronous	3 (17)	7 (23)	3 (27)	1 (100)		
	Unknown Primary	6 (33)	7 (23)	4 (36)	0 (0)		
#N LM (median [IQR])		1.0 [1.0, 3.0]	3.0 [2.0, 4.8]	1.0 [1.0, 5.0]	1.0 [1.0, 1.0]	0.071	9.1
Diameter LM (median [IQR])		6.4 [2.5, 9.5]	4.0 [2.1, 9.4]	2.1 [0.9, 4.0]	13.2 [13.2, 13.2]	0.17	24.7
Extrahepatic disease (%)	Yes	2 (11)	3 (10)	3 (27)	0 (0)	0.499	5.2
Local Therapy LM (%)	Resection	15 (88)	22 (81)	9 (82)	1 (100)	0.901	15.6
	Resection + Ablation	2 (12)	5 (19)	2 (18)	0 (0)		
Mitoses LM(%)	< 2 per 10 HPF	4 (44)	5 (45)	2 (67)	0 (NaN)	0.956	66.2
	2–20 per 10 HPF	4 (44)	5 (45)	1 (33)	0 (NaN)		
	> 20 per 10 HPF	1 (11)	1 (9)	0 (0)	0 (NaN)		
Ki67index LM(%)	< 3%	10 (67)	13 (50)	3 (43)	0 (0)	0.507	19.5
	3–20%	3 (20)	10 (38)	4 (57)	1 (100)		
	> 20%	2 (13)	3 (12)	0 (0)	0 (0)		
Grade LM (%)	Low (G1)	7 (44)	11 (37)	3 (30)	0 (0)	0.934	9.1
	Intermediate (G2)	7 (44)	12 (40)	4 (40)	1 (100)		
	High (G3), small cell	2 (12)	6 (20)	3 (30)	0 (0)		
	High (G3), large cell	0 (0)	1 (3)	0 (0)	0 (0)		
Differentiation LM (%)	Well Differentiated	8 (100)	13 (93)	2 (67)	0 (NaN)	0.901	59.7
Liver resection (%)	R0	7 (100)	11 (85)	4 (100)	1 (100)	0.571	63.6
Recurrence (%)	No	11 (61)	22 (71)	6 (60)	1 (100)	0.751	5.2
Extrahepatic recurrence (%)	No	5 (36)	13 (62)	3 (43)	1 (100)	0.56	33.8
	Yes	4 (29)	6 (29)	2 (29)	0 (0)		
	Primary not resected	2 (14)	0 (0)	0 (0)	0 (0)		
	Unknown Primary	3 (21)	2 (10)	2 (29)	0 (0)		
Location recurrence (%)	Liver	10 (100)	21 (95)	6 (86)	1 (100)	0.466	39
	Lymph Nodes	0 (0)	1 (5)	0 (0)	0 (0)		
	Peritoneum	0 (0)	0 (0)	1 (14)	0 (0)		
Location extrahepatic disease (%)	Brain	0 (0)	1 (20)	0 (0)	0 (NaN)	NaN	85.7
	Bone	1 (100)	2 (40)	1 (50)	0 (NaN)		
	Lungs	0 (0)	1 (20)	0 (0)	0 (NaN)		
	Peritoneum	0 (0)	1 (20)	0 (0)	0 (NaN)		
	Distal Lymph Nodes	0 (0)	0 (0)	1 (50)	0 (NaN)		
Solitary recurrence (%)	Solitary	4 (40)	14 (67)	4 (57)	1 (100)	0.439	40.3
Treatment intent for recurrent disease (%)	Curative	4 (50)	5 (26)	6 (67)	1 (100)	0.128	40.3
Year of treatment	2001–2011	12 (66.7)	17 (58.6)	6 (54.5)	0 (0.0)	0.579	4.1
	2012–2021	6 (33.3)	12 (41.4)	5 (45.5)	1 (100.0)		

*CCI* Charlson Comorbidity index, *CTx* Chemotherapy, *PT* Primary Tumor, *LM* Liver metastases

Treatment of patients over time was distributed equally between the time intervals of 2001–2011 and 2012–2021.

Table 2 details the perioperative treatment per HGP. There was no significant difference in perioperative treatment per HGP either. Somatostatine analogues were the most commonly used form of systemic therapy. However, only a minority of patients with liver metastases underwent neoadjuvant or adjuvant systemic therapy.

**Table 2:** Neoadjuvant and adjuvant treatment for primary tumor and liver metastases per HGP

	dHGP	pHGP	rHGP	p	Missing
n	18	33	11		
Neoadjuvant primary					
PRRT	0 (0)	0 (0)	1 (9)	Na	9.1
Somatostatin analogue	1 (6)	8 (24)	2 (18)		
Systemic chemotherapy	0 (0)	0 (0)	0 (0)		
adjuvant primary					
PRRT	0 (0)	0 (0)	0 (0)	Na	13
Somatostatin analogue	0 (0)	5 (16)	2 (18)		
Systemic chemotherapy	2 (11)	0 (0)	0 (0)		
Neoadjuvant liver metastases					
PRRT	0 (0)	1 (3)	0 (0)	0.765	5.2
Somatostatin analogue	2 (11)	7 (21)	2 (18)		
Systemic chemotherapy	0 (0)	1 (3)	1 (9)		
Adjuvant liver metastases					
PRRT	1 (6)	0 (0)	1 (9)	0.168	15.6
Somatostatin analogue	2 (12)	5 (16)	1 (9)		
Systemic chemotherapy	2 (12)	0 (0)	0 (0)		

*PRRT* Peptide Receptor Radeonucleotide Therapy

The median follow-up for survivors was 94 months (IRQ: 58–139 months). During the follow-up, 40 patients (63%) developed recurrent disease, with 22 (35%) having recurrent disease limited to the liver. There were 16 patients (40% of the patients with recurrence) who underwent curative intent local treatment for recurrent metastatic disease. The median time to recurrence was 50 months (IQR: 23-not reached).

Five-year OS was 76% (95% CI: 66–87%) for the overall cohort. For predominant dHGP, 5-year OS was 92% (95% CI: 77–100%), for predominant pHGP, 5-year OS was 73% (95% CI: 59–91%), for predominant rHGP, 5-year OS was 50% (95% CI: 25–100%). The single patient with mixed HGP had no event for OS during follow-up.

Five-year DFS was 39% (95% CI: 19–83%) for predominant dHGP, 44% (95% CI: 27–71%) for predominant rHGP and 50% (95% CI: 23–100%) for predominant pHGP. One patient with mixed HGP had no event for DFS.

**Fig. 2:**
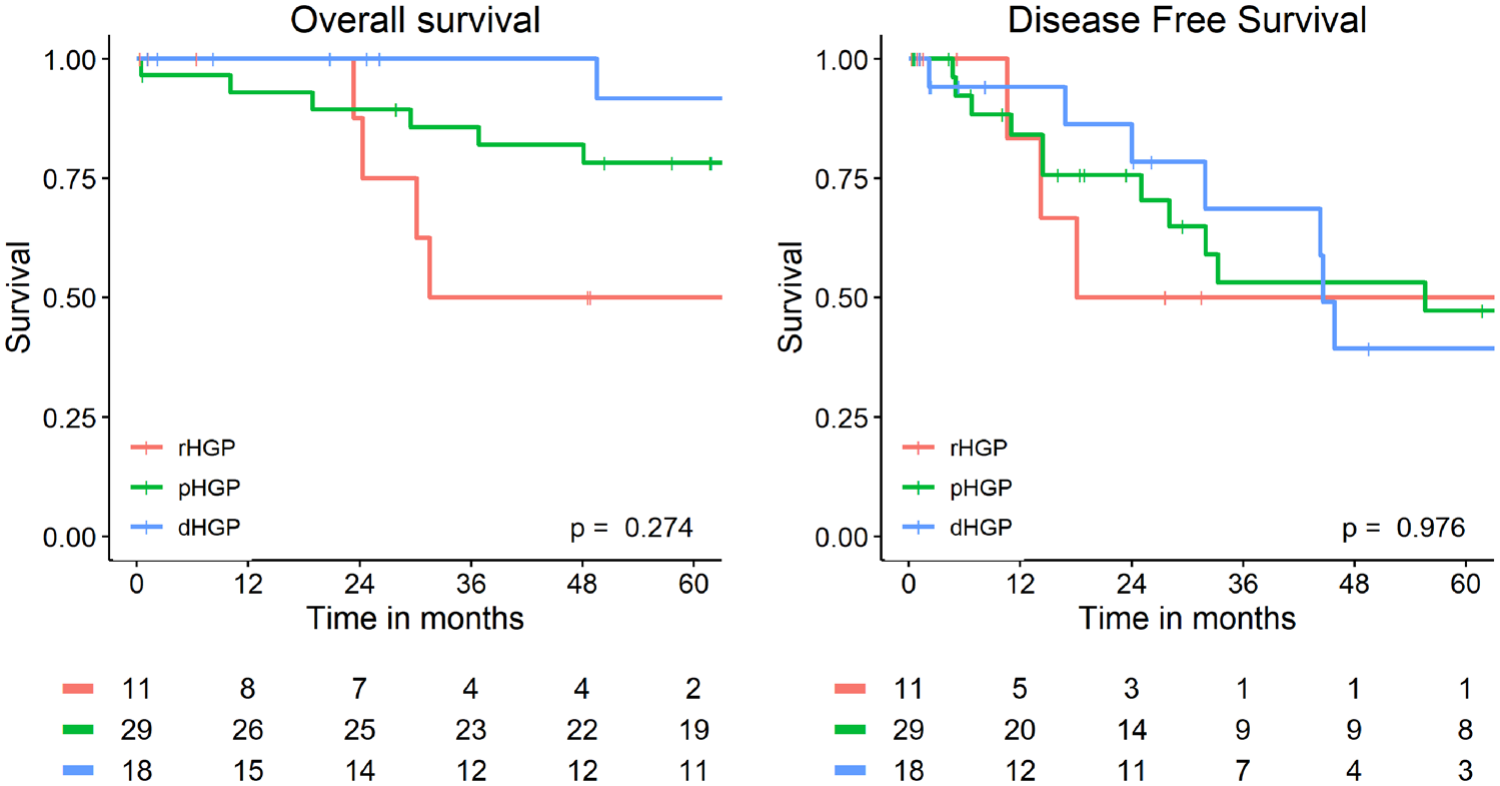
Overall and Disease Free Survival per predominant HGP group

The Kaplan–Meier curves for OS and DFS are shown in Fig. 2.

Multivariable cox regression showed no significant association between predominant HGP and OS or DFS. The only significant predictor for OS in multivariable analysis was tumor grade 3, HR 4.43 [95% CI 1.07–18.29], p = 0.04. The results of the uni- and multivariable cox regression for OS are shown in Table 3. There were no statistically significant predictors for DFS in multivariable analysis. The results of the uni- and multivariable cox regression for OS are shown in Table 4.

**Table 3:** Uni- and multivariable cox regression for Overall survival

Overall survival	Univariable	p	Multivariable	p
	HR [95% CI]		HR [95% CI]	
Predominant HGP				
Desmoplastic	Reference	–	Reference	–
Pushing	1.10 [0.37–3.24]	0.86	0.78 [0.22–2.73]	0.7
Replacement	2.62 [0.68–10.10]	0.16	3.45 [0.73–16.41]	0.12
WHO Grade				
Grade 1	Reference	–	Reference	–
Grade 2	2.22 [0.79–6.24]	0.13	2.55 [0.74–8.79]	0.14
Grade 3	2.95 [0.84–10.31]	0.09	2.96 [0.56–15.62]	0.2
Origin Primary Tumour				
GEP	Reference	–	Reference	–
Unknown primary	0.52 [0.17–1.57]	0.24	0.58 [0.15–2.30]	0.44
Lung	1.93 [0.70–5.38]	0.21	1.69 [0.32–8.97]	0.54

GEP NET is chosen as reference because it is the most common

Limited number of covariates is due to limited number of events (n = 19)

1 case of mixed HGP (50% dHGP/ 50% rHGP) excluded from analysis

**Table 4:** Uni- and multivariable cox regression for Disease-free survival

Disease free survival	Univariable	p	Multivariable	p
	HR [95% CI]		HR [95% CI]	
Predominant HGP				
Desmoplastic	Reference	–	Reference	–
Pushing	1.01 [0.42–2.42]	0.98	1.06 [0.39–2.86]	0.91
Replacement	1.15 [0.30–4.39]	0.83	1.29 [0.29–5.71]	0.74
WHO Grade				
Grade 1	Reference	–	Reference	–
Grade 2	1.07 [0.50–2.29]	0.87	1.29 [0.55–3.02]	0.56
Grade 3	0.15 [0.02–1.16]	0.07	0.21 [0.02–1.75]	0.15
Origin Primary Tumour				
GEP	Reference	–	Reference	–
Unknown primary	1.49 [0.67–3.33]	0.33	0.83 [0.30–2.25]	0.71
Lung	2.05 [0.72–5.84]	0.18	0.80 [0.19–3.32]	0.76

GEP NET is chosen as reference because it is the most common

Limited number of covariates is due to limited number of events (n = 19)

1 case of mixed HGP (50% dHGP/ 50% rHGP) excluded from analysis

## Discussion

This retrospective multicenter cohort study described the HGPs of resected NETLM. There was a high prevalence of pushing HGP, compared to the proportions of HGPs described in colorectal-, breast, or melanoma liver metastases [1, 3, 6, 7]. Multivariable analysis showed no association between HGP and OS or DFS in patients with NETLM.

NETs are a heterogeneous group of tumors with varying clinical course. Liver metastases are common, occurring in up to one third of patients with a NET [9]. The clinical course of the disease is often indolent, even in when metastases have occurred [9, 18]. Curative intent local treatment, consisting of resection sometimes combined with ablation, of metastatic lesions has become the standard of care for NETLM. Local treatment may be indicated for patients with liver-only metastatic disease and a select number of patients with limited extrahepatic disease [9, 11].

Surgical debulking in case of irresectable liver disease or extensive extrahepatic metastatic spread is generally reserved for symptom reduction in patients with functioning NETs or to alleviate mechanical complications like obstruction [9, 11, 19].

Limited biomarkers are available to stratify local treatment [9, 11, 19]. The biomarkers that are currently in use to stratify patients for local treatment are tumour grade and Ki-67 index Novel biomarkers in patients who are eligible for local treatment may enable prospective clinical trials to identify the patients that benefit most from local treatment of NETLM.

HGPs are a prognostic and possibly predictive biomarker in patients with liver metastases [1, 5]. HGPs have been studied in liver metastases from various primary tumors including colorectal cancer, breast cancer, and melanoma [3, 6, 7]. These studies, among others, have resulted in consensus guidelines for scoring HGPs in malignant liver tumors [1]. DHGP is associated with favourable prognosis with regards to OS and DFS after resection as compared to replacement or pushing HGP in all evaluated tumor-types. However, there appears to be a tumor-type specific cut-off with regards to how much dHGP has to be present at the TLI in order to affect patient prognosis [1, 6]. Pushing HGP is the least common growth pattern, occurring, for example, in less than 2% of patients with CRLM. Therefore, not much is known about the prognostic significance of pHGP [1].

In this perspective, the documentation of HGPs in NETLM could provide new insights in this disease, to better understand its biological course, to improve the individual prognostication and, ultimately, to improve the therapeutic approach. To our knowledge, the current study is the first evaluation of HGPs in NETLM. The most remarkable finding was the relatively high prevalence of pushing HGP in NETLM, as compared to the proportions of pHGP observed in colorectal-, breast, or melanoma liver metastases.[1, 3, 6, 7]. In the current cohort, pHGP was the most common HGP found at the TLI. This observation is in line with the apparent tumor-type related variation of HGPs [1]. There are multiple potential explanations for the high prevalence of pushing HGP. Previous studies in CRLM have described increased intra-and peritumoral immune infiltration in dHGP compared to non-dHGP in CRLM. The immune infiltrate in dHGP is characterize by increased absolute and relative numbers of cytotoxic CD8 + cells [20, 21]. These findings suggest increased cytotoxic anti-tumor activity in dHGP. Neuroendocrine tumors generally show a cold tumor immune microenvironment characterized by scarce lymphocyte infiltration. This lack of immune activity is further supported by the mostly poor response of NETs to immunotherapy, except for a select minority of patients who do respond well [22, 23]. The relative absence of dHGP in these immunologically cold tumors supports the hypothesis that anti-tumor immune activation may be an important factor in the origin of dHGP.

A second potential explanation for the high prevalence of pHGP in NETLM is related to another hypothesis for the biological mechanisms behind HGPs. This hypotheses for the origin of HGPs is that they reflect two different response patterns to liver injury [1, 16]. Liver injury can result in two distinct patterns, such as fibrosis or regeneration [24]. Previous studies have shown similarities between the desmoplastic rim in dHGP and fibrosis as a response to liver injury. Similarly, the replacement of hepatocytes by tumor cells in rHGP mimics the replacement of damaged hepatocytes by new hepatocytes in liver regeneration [16]. NETLM have a relatively indolent course with slow progression, compared to liver metastases from other primary tumors [9, 10]. The slow growth and less aggressive course of NETLM may not elicit a strong injury response in the liver, resulting in absence of fibrotic tissue formation and a pushing HGP in the slow growing lesions. DHGP and rHGP may still be present in the more rapidly growing and more aggressive lesions, respectively. In the current study, there was no statistically significant association between WHO grade or KI67 index and growth pattern, which would have supported this theory. However, a low WHO grade and KI67 index < 3% were more common in dHGP and pHGP compared to rHGP.

Lastly, perioperative treatment must be considered as a contributing factor to the HGPs as well. Previous studies in CRLM have shown that preoperative chemotherapy can alter HGPs [25]. This does not seem likely for the current study because there were no significant differences in perioperative therapy for the primary tumor or in preoperative therapy for the liver metastases between the HGP groups.

This study is limited by the small sample size. This makes comparison of HGP groups with regards to survival difficult. In the present cohort, multivariable analysis showed no association between HGP and OS or DFS in patients who underwent resection of NETLM. Yet, a tendency for better postoperative OS was observed in patients with predominant dHGP as compared to those with predominant pHGP and predominant rHGP. Broad inclusion criteria were used to keep the sample size as large as possible for analytical purposes. This also results in a heterogeneous population with regards to some of the most important predictors for survival including origin of the primary tumour and tumour grade, which makes the study prone to bias. Notably, beside the limited number of patients evaluated, the prognosis of NETLM is relatively good, leading to only few survival events, limiting the number of covariates that can be corrected for in multivariable analysis. This further compounds the problem posed by the heterogeneity in the sample. Furthermore, this study only includes patients who underwent resection for NETLM, so only patients with limited metastatic disease, mostly confined to the liver, and/or symptomatic disease in case of a functioning tumor were evaluated, which probably resulted in a selection bias [9]. The long inclusion period also provides a source of potential bias. Advances in clinical practice may have favourably affected the prognosis of patients who were treated more recently compared to patients who were treated towards the start of the inclusion period. There was no statistically significant difference in the distribution of the date of CRLM resection between the HGPs, which suggests that the potential bias is somewhat equally distributed between the HGP groups. However, due to the small number of events, it was not possible to correct for date of resection as a covariate in multivariable analysis.

With these limitations, we cannot draw definitive conclusions on the prognostic value of HGPs in NETLM based on this study. HGPs have been demonstrated to be an independent prognostic biomarker in patients with colorectal cancer liver metastases [3, 4]. As such, they may have a role as an addition to the existing biomarkers in NETLM, like tumour grade and KI-67 index. There are two major obstacles to the implementation of HGPs in NETLM in clinical practice. First is the limited availability of the data, making it difficult to assess the prognostic value of HGPs and their relationship with other known predictors of prognosis. The second difficulty with the application of HGPs as a biomarker is that they are currently only available after resection of liver metastases. Preoperative determination of HGPs is a necessary step to overcome these obstacles. Firstly, preoperative determination of HGPs would enable the inclusion of patients with NETLM who do not undergo resection in clinical studies. This could increase the sample size of studies and enable researchers to do extensive multivariable survival analyses into the prognostic value of HGPs in patients with NETLM and the association of HGPs with other known predictors of survival in this group. If HGPs do have prognostic value in NETLM, similar to their value in CRLM, then preoperative availability would allow clinicians to use HGPs as an adjunct to the currently available predictors of survival. Advances have been made in the use of radiomics and AI to preoperatively determine HGPs in patients with CRLM using routine imaging modalities, with promising preliminary results [26].

In conclusion, pushing HGP is the most common HGP in resected NETLM. This is in sharp contrast with findings in liver metastases of breast cancer, colorectal cancer and melanoma. No statistically significant association was found between HGPs and OS or DFS in NETLM. However, this analysis is limited by the relatively small sample size.

## Data Availability

The datasets generated during and/or analysed during the current study are available from the corresponding author on reasonable request.
